# The Importance of Molecular Testing in the Diagnosis of Genetic Syndromes with Chronic Kidney Disease: Genotype–Phenotype Correlations

**DOI:** 10.3390/ijms27052362

**Published:** 2026-03-03

**Authors:** Lăcrămioara Ionela Butnariu, Radu Russu, Ramona Geanina Babici, Aurora Băgiag, Laura Mihaela Trandafir, Elena Țarcă, Paula Popovici, Nicoleta Gimiga, Iuliana Magdalena Starcea

**Affiliations:** 1 Department of Medical Genetics, Faculty of Medicine, Grigore T. Popa University of Medicine and Pharmacy, 700115 Iasi, Romania; laura.trandafir@umfiasi.ro (L.M.T.); elena.tuluc@umfiasi.ro (E.Ț.); paula.popovici@umfiasi.ro (P.P.); nicoleta.chiticariu@umfiasi.ro (N.G.); magdalenastarcea@gmail.com (I.M.S.); 2 Regional Center for Medical Genetics Iasi, Saint Mary’s Emergency Children Hospital, 700309 Iași, Romania; 3 Departament of Nefrology, Saint Mary’s Emergency Children Hospital, 700309 Iași, Romania; radurussu@yahoo.com; 4 Dr. C.I. Parhon University Hospital, 700503 Iasi, Romania; 5 Department of Modern Languages, “Iuliu Haţieganu” University of Medicine and Pharmacy, 400012 Cluj-Napoca, Romania; aurora.bagiag@umfcluj.ro; 6 Department of Pediatrics, Saint Mary’s Emergency Children Hospital, 700309 Iași, Romania; 7 Department of Surgery II—Pediatric Surgery, Saint Mary’s Emergency Children Hospital, 700309 Iași, Romania

**Keywords:** chronic kidney disease, polycystic kidney disease, Alport syndrome, Bartter syndrome, MODY5, genetic testing, genetic counseling

## Abstract

Globally, chronic kidney disease (CKD) affects over 800 million individuals and is characterized by significant genetic complexity. More than 600 genes are associated with hereditary kidney disease, which may manifest as isolated kidney issues or as part of a syndrome that also includes extrarenal manifestations. The aim of this study was to identify genetic variants in a group of ten patients who presented with clinical signs suggestive of genetic syndromes associated with CKD, or who were asymptomatic but had a positive family history of CKD. Extensive genetic testing (targeted gene panels and whole-exome sequencing—WES) identified a mutation in the *PKD1* gene in 3 out of 10 cases. In one patient, a known mutation in the *PKD2* gene was identified. Another four patients were diagnosed with Alport syndrome: three of these presented with de novo missense mutations in the *COL4A5* gene, and one patient had a mutation in the *COL4A3* gene. One patient was diagnosed with MODY5, caused by a known mutation in the *HNF1B* gene, and one patient was diagnosed with Bartter syndrome type 1, resulting from a known mutation in the *SLC12A1* gene. We present genotype–phenotype correlations, highlighting the particularities of each patient within their family context. Our findings emphasize the importance of genotype–phenotype correlations in refining diagnosis, personalizing therapeutic management, and providing essential genetic counseling for at-risk relatives.

## 1. Introduction

Chronic Kidney Disease (CKD) is a significant cause of morbidity and mortality with a global prevalence exceeding 10% [1,2], affecting over 850 million people worldwide and approximately 100 million Europeans [3,4]. CKD is a progressive condition that encompasses a large number of individual entities that manifest through abnormalities in kidney structure and function [3,4,5].

**Figure 1:**
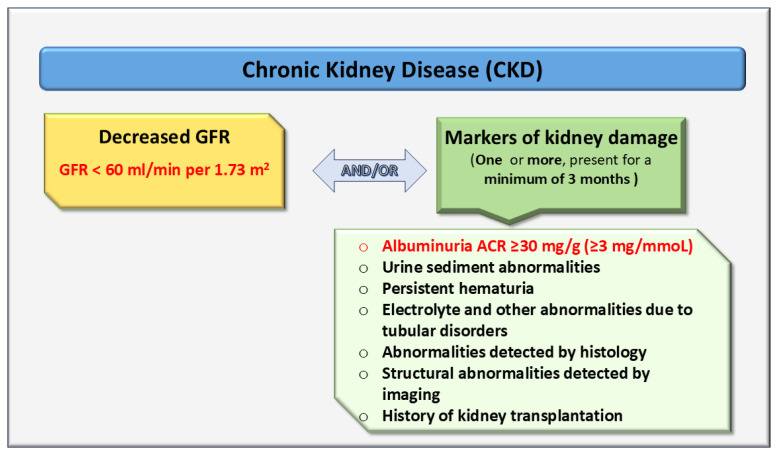
Criteria for Chronic Kidney Disease According to KDIGO [4,5]. ACR: albumin-to-creatinine ratio; GFR: glomerular filtration rate.

According to the KDIGO guidelines, CKD is characterized by structural or functional abnormalities of the kidney that persist for at least 3 months, with implications for health status. CKD is defined according to its causes, a decreased glomerular filtration rate (GFR <60 mL/min per 1.73 m^2^), an increased albumin-to-creatinine ratio (ACR ≥30 mg/g (≥3 mg/mmoL), plus additional markers of kidney damage (urinary sediment abnormalities, persistent hematuria, electrolyte disturbances due to tubular dysfunction, histological abnormalities, structural changes on imaging, and history of kidney transplantation) (Figure 1) [4,5].

Similar to other complex diseases, the heritability of CKD varies between 35% and 69% [6]. Impaired kidney function can be caused by monogenic mutations, but many kidney diseases are complex polygenic diseases resulting from the interaction of multiple genetic variants and environmental/lifestyle factors (a multifactorial etiology) [6].

In recent years, advances in genomic research and extensive genetic testing, which includes targeted gene panels, Whole-Exome Sequencing (WES) or Whole-Genome Sequencing (WGS), have improved diagnostic accuracy and prognosis and enabled personalized management of patients with CKD [3,7,8].

Currently, more than 600 monogenic kidney diseases are known, which are responsible for 50% of childhood-onset CKD; most of the causative variants are very rare or family-specific variants [3] (Table 1). Unlike in children, the prevalence of Mendelian kidney diseases is about 10% in adult patients, though this varies based on factors like kidney disease type, family history, age of onset, and other symptoms (extrarenal manifestations) [1,3].

Rare hereditary chronic kidney diseases (CKDs) present a heterogeneous etiology (Table 1) that frequently causes variable phenotypes correlated with genetic phenomena such as pleiotropy, incomplete penetrance, and variable expressivity [1,52].

Congenital anomalies of the kidney and urinary tract (CAKUT), ciliopathies (such as Autosomal Dominant Polycystic Kidney Disease, ADPKD), Alport syndrome (AS), autosomal dominant tubulointerstitial renal disease (ADTKD), and monogenic nephrotic syndromes are the leading causes of CKD in children [53,54].

ADPKD is the most common hereditary kidney disease which can be diagnosed early in patients with a positive family history, but which usually manifests in adulthood [55].

Unlike in children, about two-thirds of kidney failure in adults are caused by hypertension and diabetes mellitus, while monogenic (genetic) causes are responsible for approximately 10% to 15% of all CKD and end-stage renal disease (ESRD) [56].

Rare, highly penetrant genetic mutations (e.g., *PAX2*, *HNF1B*, *EYA1*, *SALL1*) and copy number variations (CNVs) cause most forms of CAKUT syndrome, but have also been associated with isolated (nonsyndromic) forms of kidney disease [1,57,58,59]. Based on recent literature and genomic studies, 17q12 deletions and 22q11.2 deletions are well-recognized pathogenic hotspots for CNVs that significantly cause structural renal anomalies. Single nucleotide polymorphisms (SNPs) and small insertions and deletions (indels) are correlated with the entire phenotypic spectrum of kidney disease [1,60].

In autosomal dominant polycystic kidney disease (ADPKD), rare somatic variants act as a “second hit” by introducing an additional mutation in the remaining functional copy of either the *PKD1* gene (located on chromosome 16p13.3) or the *PKD2* gene (located on chromosome 4q22.1), thereby promoting cyst formation [61,62]. This “two-hit” model—in which a germline mutation serves as the first hit and a subsequent somatic mutation as the second—also appears to drive the development of clonal hematopoiesis of indeterminate potential (CHIP). CHIP, in turn, may increase the risk of complications such as renal failure and other adverse outcomes associated with CKD [63].

Recent research has shown that mutations in the genes encoding type IV collagen (*COL43*, *COL4A4* and *COL4A5*) expressed in glomerular basement membranes are associated with various forms of kidney disease, highlighting its importance in maintaining normal kidney function. Thus, X-linked and autosomal recessive variants of Alport syndrome have been identified. Rare mosaic variants are known to influence phenotypic severity in X-linked Alport syndrome (XLAS). Thus, in male patients, the severity of the disease correlates with the proportion of affected cells (a lower percentage of cells containing the pathogenic variant tends to lead to a milder clinical phenotype) [1,64].

Syndromic Focal Segmental Glomerulosclerosis (FSGS) is a form of inherited kidney disease where FSGS occurs as one component of a broader genetic syndrome caused by mutations in genes (e.g., *WT1*, *LMX1B*, *LAMB2*) that affect both the podocytes and other organs, resulting in extrarenal manifestations (Table 1). The most common syndromes include Denys-Drash, Frasier, Nail-Patella, and Pierson syndromes, which feature abnormalities in vision, hearing, or genitalia alongside proteinuria [28,29,30,31,32]. Unlike primary (idiopathic) FSGS, these genetic forms typically present in childhood or young adulthood with steroid-resistant nephrotic syndrome and frequently require early genetic testing to guide management, as they rarely respond to immunosuppressive therapies [10,11,28,29,30,31,32].

Bardet–Biedl Syndrome (BBS) is a rare autosomal recessive ciliopathy caused by mutations in over 26 different *BBS* genes (*BBS1*–*BBS26*) that disrupt cilia function [9,22]. It requires pathogenic mutations in both copies of a gene (biallelic), often involving founder mutations (e.g., *BBS1* p.M390R, *BBS10* p.C91Lfs*5) [9]. *BBS1* and *BBS10* are the most common causes, accounting for 40–50% of cases [22,23].

Pathogenic mutations in the *TSC1* (located on chromosome 9q34.13) or *TSC2* (located on chromosome 16p13.3) genes cause tuberous sclerosis complex (TSC), a rare genetic disorder characterized by the growth of benign tumors (hamartomas) in various organs (brain, kidneys, heart, eyes, lungs and skin) [24,25]. While both genes are involved in regulating cell growth and division, *TSC2* pathogenic variants cause a more severe phenotype—including a higher risk of renal angiomyolipomas, renal malignancy, and cognitive impairment—compared to *TSC1* mutations. Symptomatic treatment includes anticonvulsant drugs, mTOR inhibitors, surgery, and patient monitoring [24,25].

Bartter syndrome is a group of rare, primarily autosomal recessive genetic disorders caused by mutations in genes (*SLC12A1*, *KCNJ1*, *CLCNKB*, *BSND*, or *MAGED2*) that regulate salt reabsorption in the kidney’s loop of Henle (Table 1). This impairs ion transport, resulting in severe salt wasting, hypokalemia, and metabolic alkalosis [9,36,37,38,39,40].

Gitelman syndrome (GTLMNS) is the most common renal tubular disorder in Caucasians (prevalence 1 in 40,000), caused primarily by inactivating mutations in the *SLC12A3* gene (located on chromosome 16q13), which encodes the thiazide-sensitive NaCl cotransporter (NCC) in the distal convoluted tubule of the kidney [9,38,39,40,41,42,43,44]. It is an autosomal recessive, salt-wasting disorder characterized by hypokalemic metabolic alkalosis, hypomagnesemia, and hypocalciuria. Clinical manifestations include periods of muscle weakness, tetany, abdominal pain, and chondrocalcinosis. It often presents in adolescence or adulthood, but cases have been reported in younger children [9,38,39,40,41,42,43,44].

Cystinuria is a genetic metabolic disorder caused by mutations in the *SLC3A1* (located on chromosome 2p21) or *SLC7A9* (located on chromosome 19q13.1) genes, which impair amino acid reabsorption in the proximal tubule of the kidney leading to painful, recurrent cystine nephrolithiasis. It is primarily inherited in an autosomal recessive pattern, though some forms show incomplete dominance [45,46,47].

Clinical forms of cystinosis are caused by autosomal recessive mutations in the *CTNS* gene (located on chromosome 17p13.2) [9] that interfere with the transport of the amino acid cystine out of lysosomes. These determine the accumulation of intracellular cystine crystals, causing extensive damage to tissues and organs, especially the kidneys and eyes [9,48]. Over 140 mutations have been reported in the literature. Large deletions (including the 57 kb deletion, which is the most common in Northern European and North American populations, accounting for 50–75% of pathogenic alleles) and truncating *CTNS* pathogenic variants (e.g., W138X) are typically associated with the severe, infantile nephropathic form of the disease [9,48]. Missense variants and splicing mutations often allow for some residual protein function, leading to milder, late-onset or juvenile forms of cystinosis or non-nephropathic (ocular) cystinosis [9,48].

Fabry disease is a rare, hereditary, X-linked lysosomal storage disorder caused by a mutation in the *GLA* gene (located on chromosome Xq22.1), which results in a deficiency of the enzyme alpha-galactosidase A (α-Gal A), leading to progressive lysosomal deposition of globotriaosylceramide and its derivatives in cells throughout the body [49]. It causes severe, multi-system damage, including neuropathic pain, skin lesions (angiokeratoma), cardiovascular and renal failure. Treatments include Enzyme Replacement Therapy (ERT) and chaperone therapy [49,50].

Pathogenic mutations in *AGXT* gene (located on chromosome 2q37.3) cause primary hyperoxaluria type 1 (PH1), a rare, autosomal recessive disorder characterized by a deficiency of the hepatic peroxisomal enzyme alanine-glyoxylate aminotransferase (AGT). Over 190 different mutations have been described that cause excessive oxalate production, leading to recurrent nephrolithiasis, nephrocalcinosis, and often end-stage renal disease (ESRD) [51].

Recent advances in genomic research have significantly transformed the landscape of CKD management, enabling a deeper and more personalized understanding of the molecular mechanisms that determine renal pathophysiology. By correlating genetic profiles with clinical data, physicians can now leverage improved diagnostic accuracy, tailor therapeutic interventions, and provide more precise genetic counseling [65].

Thus, recent clinical guidelines, including KDIGO (Kidney Disease Improving Outcomes), emphasize the importance of genetic testing in the diagnosis and management of patients with CKD [65,66].

This retrospective study aims to analyze and identify pathogenic genetic variants in a group of patients diagnosed with genetic syndromes associated with chronic kidney disease (CKD). We also presented genotype–phenotype correlations, highlighting the particularities of each patient and their family, in relation to the identified genetic variant. These targeted the spectrum of clinical manifestations (disease severity, age of onset and progression rate to CKD), management strategies (including family screening and genetic counseling) and personalized treatment.

## 2. Results

**Figure 2:**
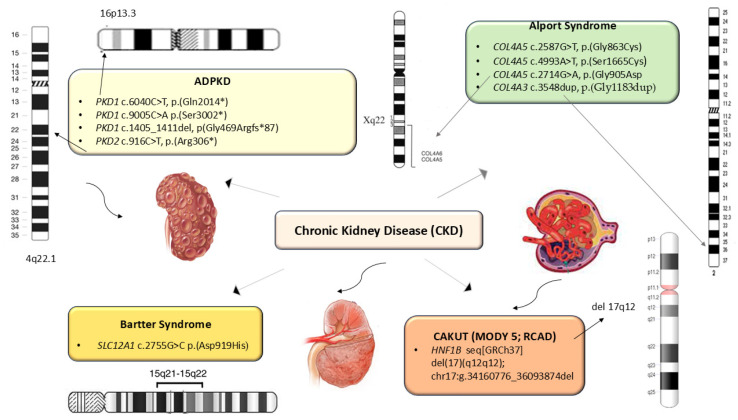
Genetic Variants Identified in Patients with Genetic Syndromes Associated with CKD. ADPKD: Autosomal dominant polycystic kidney disease; CAKUT: Congenital anomalies of the kidney and urinary tract; MODY5: Maturity-Onset Diabetes of the Young type 5; RCAD: Renal Cysts and Diabetes Syndrome.

Extensive genetic testing (targeted gene panels that included genes for CKD and WES) in ten patients who had clinical signs specific to genetic syndromes that associate CKD or asymptomatic patients who had a positive family history for CKD, identified in 3/10 cases a mutation in the *PKD1* gene (Table 2). In 1/10 patients a known mutation in the *PKD2* gene was identified; another 4/10 patients were diagnosed with Alport syndrome: 3/10 patients presenting de novo missense mutations in the *COL4A5* gene and 1/10 patients a variant in the *COL4A3* gene; 1/10 patients were diagnosed with MODY5 caused by a known mutation in the *HNF1B* gene, and 1/10 patients were diagnosed with Bartter syndrome type 1, caused by a known mutation in the *SLC12A1* gene (Table 2 and Figure 2).

Nonsense variants were found in three of the patients with polycystic kidney disease: *PKD1* c.6040C>T, p.(Gln2014*) (P01), *PKD1* c.9005C>A p.(Ser3002*) (P02) and *PKD2* c.916C>T, p.(Arg306*) (P04) (Table 2 and Figure 2).

Hemizygous missense variants in the *COL4A5* gene were identified in two patients with X-linked AS (XLAS): c.4993A>T (P06) and c.2714G>A (P07). Patient P05 (L.E, female) was diagnosed with XLAS caused by a likely pathogenic heterozygous mutation in the *COL4A5* (c.2587G>T) of paternal origin. Another patient (P08, C.C.) with AS with autosomal dominant inheritance presented a de novo heterozygous duplication in the *COL4A3* (c.3548dup) (Table 2 and Figure 2).

Other variants detected in CKD patients included a large heterozygous deletion involving the *HNF1B* gene identified in a patient with Maturity-Onset Diabetes of the Young type 5 (MODY5) (P09) and a known missense mutation in the *SLC12A1* that caused Bartter syndrome type 1 in another patient (P10) (Table 2 and Figure 2).

**Figure 3:**
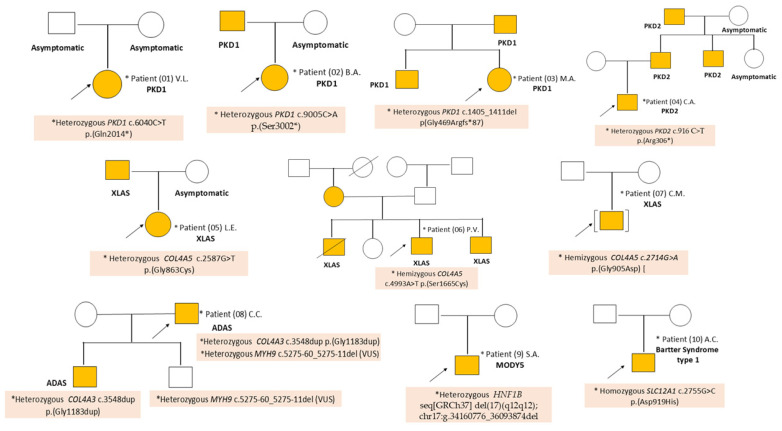
Family Trees and Data Collected in the Case of Patients with Genetic Syndromes Associated with CKD.

Family history was positive for a genetic syndrome associated with CKD in 6/10 patients: 3/10 patients with autosomal dominant polycystic kidney disease (ADPKD) (P02, P03 and P04) and 3/10 patients with Alport syndrome (P05, P06 and P08); in the case of patient (P09) with MODY5 and (P10) with Bartter syndrome there were no other affected individuals in their families (Figure 3).

The clinical data of the ten patients included in the study are presented in Table 3.

The age of the patients at the time of diagnosis ranged between 17 months and 47 years, and most patients were male (7 out of 10 patients). All patients were dynamically monitored, following markers of renal damage and the decrease in glomerular filtration rate (Table 3).

## 3. Discussion

Three known mutations in the *PKD1* (P01 and P02) and *PKD2* (P04) genes, and one novel mutation in the *PKD1* gene (P03), were identified in patients with ADPKD (Table 1).

### 3.1. Genotype–Phenotype Correlations in Autosomal Dominant Polycystic Kidney Disease

ADPKD is the most common inherited kidney disease (with a prevalence of 1 in 400 to 1000 individuals) [62,67], manifested by fluid-filled cysts in kidneys and other organs (liver, pancreas, brain, heart), and is caused by mutations in the *PKD1* (85%), and *PKD2* (15%) genes. Symptoms of the disease usually appear in adulthood (30–40 years), and the evolution is progressive; the growth of cysts causes kidney enlargement, pain, hypertension, infections and, finally, end-stage-renal disease (ESRD). Disease management focuses on symptomatic treatment that includes antihypertensive medication, diet, hydration, and newer therapies like mTOR inhibitors (rapamycin analogs), somatostatin analogs, and tyrosine kinase inhibitors to slow disease progression and preserve kidney function [68,69,70].

Male gender is recognized as a risk factor for faster kidney cyst growth and progression to ESRD, suggesting that androgens might promote it, while estrogen might be protective [62].

Although *PKD1* and *PKD2* cause a similar phenotype, *PKD1* variants are associated with more severe disease and earlier ESRD (around age 53), compared to *PKD2* mutations, in which the disease progression is less severe, with fewer renal cysts, and late-onset hypertension and ESRD (around age 72) [71,72,73].

Atypical ADPKD are associated with mutations in the *GANAB*, *DNAJB11*, *IFT140*, and other genes [70,71,72,73]. *GANAB* mutations are associated with a milder phenotype but with more variable polycystic liver disease [62]. Other variants in the *PRKCSH*, *SEC63*, *LRP5*, *ALG8*, and *SEC61B* genes cause autosomal dominant polycystic liver disease (ADPLD) which can be associated with mild kidney disease [74,75].

To date, a large number of *PKD1* and *PKD2* variants have been reported. Thus, *PKD1* nonsense, frameshift, or splice-site alterations associated with truncated protein mutations are more severe than those not associated with truncated protein mutations (e.g., missense and in-frame insertion/deletion mutations) Most are unique to individual families, making genetic screening crucial for early diagnosis [62,75].

*TSC2/PKD1* contiguous gene deletion syndrome (also known as Polycystic Kidney Disease with Tuberous Sclerosis, or PKDTS) is a rare, severe disorder resulting from a contiguous deletion on chromosome 16p13.3 [9,24]. Patients present with aggressive, early-onset renal cystic disease, which can be diagnosed prenatally or in early childhood. Together with renal cysts, renal angiomyolipomas lead to rapid progression to renal failure, typically in the first three decades of life [75,76,77,78].

The first patient (P01, V.L), a 1-year-and-10-month-old diagnosed with stage I CKD, had a clinical history notable for recurrent urinary tract infections (UTIs), anemia, and hepatocytolysis. Although his family history was negative for kidney disease, a renal ultrasound showed bilateral renal cystic formations. Through extended sequencing with the Blueprint Genetics (BpG) Cystic Kidney Disease Panel, a heterozygous pathogenic nonsense variant was identified in the *PKD1* gene (c.6040C>T, p.(Gln2014*)), as shown in Table 1.

This variant is absent from Genome Aggregation Database (gnomAD) [79] but documented in ClinVar database (variation ID 1253802) [80]. This mutation introduces a premature stop codon in exon 15, likely causing nonsense-mediated mRNA decay or a truncated protein [81]. Loss-of-function (LoF) is an established disease mechanism for *PKD1* [81]; this specific variant has been reported in multiple patients with ADPKD (PMID: 12007219, 25646624, 22508176) [82,83,84].

The second patient (P02, B.A.) was initially evaluated at 17 months of age. Although she was asymptomatic at the time, genetic testing was recommended due to her positive family history of ADPKD (her father had renal and hepatic cysts). In this case, the diagnosis of renal disease was made at a presymptomatic stage. Sequence analysis using the Blueprint Genetics (BpG) Cystic Kidney Disease Panel identified a heterozygous nonsense variant, *PKD1* c.9005C>A p.(Ser3002*), which is classified as likely pathogenic and associated with polycystic kidney disease (Table 1).

This variant, a nonsense mutation in exon 25 (out of 46) of the relevant gene, was previously reported by Pinto et al. (PMID: 37353797) [85]. It introduces a premature stop codon, which is predicted to result in a loss of protein function [85]. The variant is listed in ClinVar (Variation ID: 1707241) as a likely pathogenic or pathogenic variant associated with a clinical presentation of renal cysts [80]. For patient P02, periodic monitoring of renal function was recommended. This includes biochemical tests (serum and urine) and renal ultrasound, as well as screening of at-risk family members through genetic testing and nephrological evaluation to detect other asymptomatic individuals.

Patient P03, M.A., was diagnosed at the age of 13 with stage I CKD. He presented with a positive family history for ADPKD: his father has polycystic kidney disease and secondary hypertension, and his 4-year-old brother has renal cysts; neither has undergone genetic testing. A renal ultrasound revealed several bilateral cortical cysts. The patient experienced an episode of acute pyelonephritis, developed secondary hypertension, and presented with proteinuria. His clinical evolution was favorable under specific treatment, and recommendations included monitoring of blood pressure values and avoidance of nephrotoxic drugs. Genetic testing using the Blueprint Genetics (BpG) Cystic Kidney Disease Panel identified a novel likely pathogenic heterozygous frameshift variant in *PKD1* c.1405_1411del, p.(Gly469Argfs*87) (Table 1).

This variant is reported to be absent from population databases such as gnomAD. It consists of a 7-base pair deletion (c.1405_1411del) located in exon 7 of the *PKD1* gene, which results in a frameshift (p.Gly469Argfs*87) and the introduction of a premature stop codon, ultimately leading to loss of protein function. The identification of variants in the first 32 exons of PKD1 is often challenging due to their high homology with pseudogenes (PKD1P1–P6). This requires specialized, high-quality sequencing techniques to accurately differentiate the true gene from its pseudogenes.

The *PKD1* variant c.1405_1411del, p(Gly469Argfs*87) was classified as likely pathogenic based on the established association between the gene and the patient’s phenotype, the variant’s absence of in control population, and variant type (frameshift).

Considering that loss-of-function mutations in *PKD1* are predictive of a more aggressive disease course and a faster decline in estimated glomerular filtration rate (eGFR), periodic assessment of renal function was advised for the three patients (P01, P02, and P03) to prevent complications.

Patient P04 (C.A.) was diagnosed by ultrasound at the age of 15 years with horseshoe kidneys and polycystic kidney disease, in the context of a positive family history: the child’s father, one of his siblings and his paternal grandfather had renal cysts (Figure 3).

Clinical manifestations included proportionate dwarfism (height = −3.04 SD, weight = −2.04 SD) and mild facial dysmorphism. Endocrinological evaluation, hormonal balance (growth and thyroid hormones) and hand-wrist radiography for bone age assessment were all within normal limits, with no indications for growth hormone therapy. In this case, monitoring of blood pressure values and periodic evaluation of renal function were also recommended. Genetic testing using the Blueprint Genetics (BpG) Cystic Kidney Disease Panel identified a pathogenic heterozygous nonsense *PKD2* c.916C>T, p.(Arg306*) variant (Table 2).

This variant introduces a premature stop codon in exon 4 (out of a total of 15 exons) and is predicted to lead to loss of normal protein function [81]. It is classified as pathogenic/likely pathogenic in ClinVar (Variation ID 397507) [80] and is documented in HGMD [81]. The *PKD2* c.916C>T, p.(Arg306*) is a well-documented pathogenic variant associated with ADPKD type 2 and has been described several times in the literature (PMID: 9326320, 24113780, 29529603, 29633482, 31740684, 33437033) [86,87,88,89,90,91].

The *PKD2* c.916C>T, p.(Arg306)* variant was previously reported by Kim et al. [90] and Mallawaarachchi et al. [91]. Both studies emphasize the critical role of genetic diagnosis in polycystic kidney disease, whether through targeted sequencing of *PKD1* and *PKD2* or via comprehensive genomic approaches like Whole-Exome Sequencing (WES) and Whole-Genome Sequencing (WGS) [90,91].

Riccio et al. [87] identified the *PKD2* c.916C>T, p.(Arg306*) variant in a 31-year-old patient who had been diagnosed with Marfan syndrome at the age of 26, inherited from his father. The patient presented with gross hematuria and abdominal pain [87]. Contrast-enhanced computed tomography of the abdomen revealed an aneurysm of the abdominal aorta and hepatic artery, along with multiple hepatic and bilateral renal cysts, suggestive of autosomal dominant polycystic kidney disease (ADPKD). Although the family history was negative for ADPKD, the patient’s mother had suffered a premature death at age 38 due to a ruptured dissecting thoracic aortic aneurysm.

The coexistence of ADPKD and Marfan syndrome is rare, with only a few cases documented in the literature [92,93]. While renal cysts represent the primary manifestation of ADPKD, extrarenal cystic involvement and connective tissue abnormalities—such as arterial aneurysms—may also occur [94]. These findings have led some researchers to propose that a generalized connective tissue defect may play a central role in the pathogenesis of ADPKD [95].

*COL4A5* gene (located on chromosome Xq22.3) (OMIM 303630) [9] encodes a distinct alpha 5 chain for the collagen type IV, a major structural component of the glomerular basement membrane together with chains 3 and 4 of collagen IV [9]. Classically, pathogenic variants in the *COL4A5* gene have been associated with X-linked Alport syndrome (XLAS) (OMIM 301050) [9]. The phenotype is variable and includes progressive renal failure due to glomerulopathy associated with sensorineural hearing loss (SNHL) and ocular anomalies [96].

Impaired hearing is frequently detectable by late childhood or early adolescence in boys with XLAS. SNHL develops in 80–90% of males with XLAS by age 40 years. In females with XLAS, hearing loss is less frequent and tends to occur later in life (GeneReviews NBK1207, PMID 37122389) [96,97]. Early-onset aortic disease may be an unusual feature of AS, and has been mainly described in patients with truncating variants (PMID 20494893) [98].

Alport syndrome has an estimated prevalence of approximately 1 in 50,000 live births [9]. The most common form is X-linked inheritance, caused by pathogenic variants in the *COL4A5* gene (OMIM 303630), which accounts for about 65% of cases. The remaining cases are attributable to autosomal recessive or dominant variants in either the *COL4A3* (OMIM 203780) or *COL4A4* (OMIM 104200) genes [9,96].

Although X-linked disorders typically present with more severe manifestations in hemizygous males, heterozygous females carrying recessive or X-linked variants may also experience significant clinical involvement. Moreover, in females with the autosomal dominant form, disease progression tends to be slower compared to the rapid decline often seen in affected males [96].

In XLAS associated with *COL4A5*, distinct genotype–phenotype correlations have been observed. Patients carrying truncating variants or large rearrangements face a substantially higher risk of ESRD, with 70% to 90% progressing to ESRD by age 30. In contrast, individuals with pathogenic missense variants have a more favorable prognosis, with the risk decreasing to approximately 50% by the same age [96].

The severity and progression of hearing loss are also influenced by the underlying genotype. Patients with truncating variants have a 50% risk of developing deafness by age 10—a progression twice as rapid as that seen in patients with missense variants, who reach the same risk threshold at age 20 [96].

Current genomic databases reflect the broad mutational spectrum underlying Alport syndrome. ClinVar lists over 700 pathogenic variants, including 30% missense, 15% frameshift, 10% splice-site, and 6% nonsense alterations [80]. The HGMD Professional database (v.2023.1) identifies more than 1330 disease-causing variants, with truncating mutations (nonsense, frameshift, and splicing) accounting for 45% of cases, followed by missense mutations (41%) and gross deletions (11%) [81].

Patient P05 (L.E., female) underwent nephrological evaluation at the age 2 years, presenting with proteinuria (microalbuminuria) and macroscopic hematuria. During follow-up, persistent hematuria was accompanied by recurrent respiratory infections and an episode of acute pyelonephritis. At her most recent evaluation (age 10), an audiogram confirmed normal hearing. Given the positive family history—specifically, her father’s diagnosis of Alport syndrome, which required a renal transplant (although he was never genetically tested)—genetic testing was recommended.

Sequence analysis utilizing the Blueprint Genetics Alport Syndrome Panel identified a heterozygous, likely pathogenic missense variant in the *COL4A5* gene [c.2587G>T, p.(Gly863Cys)] (Table 1). This specific variant is absent from the gnomAD database and has not been previously documented in medical literature or disease-specific variation databases. However, multiple in silico prediction tools suggest a deleterious effect. The variant involves a highly conserved glycine residue within the triple helical domain of this fibrillar collagen; according to the Human Gene Mutation Database (HGMD) [81], the substitution of such glycine residues is a well-established pathogenic mechanism in Alport syndrome [81]. Furthermore, a different substitution at the same codon [c.2587G>A, p.(Gly863Ser)] is listed in ClinVar (ID: 635542) as associated with *COL4A5*-related phenotypes, further supporting the critical functional importance of this amino acid position [80].

Due to skewed X-inactivation, heterozygous females with XLAS present with a wide range of clinical phenotypes [96]. Consequently, our patient’s follow-up was comprehensive, including nephrological care as well as regular ophthalmological assessments and audiograms, since hearing loss typically occurs later in females compared to males with XLAS [96].

Patient P06 (P.V.), a 26-year-old male, presented with end-stage renal disease (CKD Stage V, on hemodialysis) and sensorineural hearing loss requiring hearing aids since childhood. A significant family history suggestive of X-linked Alport Syndrome (XLAS) was noted: his mother and two brothers had CKD requiring dialysis, and a sister had microscopic hematuria; however, none had undergone prior genetic testing.

The combination of early-onset hearing loss and a spectrum of renal manifestations—including acidosis, hyperkalemia, anemia, hematuria, and proteinuria—strongly supports the diagnosis of XLAS in this patient.

Genetic testing using the Illumina TruSight Cardio Sequencing Panel (Illumina, Inc., San Diego, United States of America) identified a likely pathogenic, hemizygous missense mutation in the *COL4A5* gene: c.4993A>T, p.Ser1665Cys). This specific variant is absent from the gnomAD v4.1.0 databases (both genomes and exomes). In silico predictions suggest a more severe functional impact, including disruption of the canonical splice donor site leading to a frameshift, protein truncation, and a total loss-of-function (LoF). Such LoF variants in the *COL4A5* gene are established causes of XLAS. According to ACMG classification guidelines [99], this variant is categorized as likely pathogenic.

Within the same splicing region, another pathogenic missense variant, *COL4A5* NM_033380.3(COL4A5):c.4994G>A (p.Ser1665Asn) (ClinVar Variation ID: 1443814) [80], exhibits computational predictions similar to those of the variant identified in the present study. This missense change has been previously documented in individuals with clinical features of Alport syndrome (PMID: 8940267, 22921432; Invitae Laboratory) [100,101].

Additionally, Wang et al. [100] identified two missense mutations—c.2777G>T (p.Gly926Val) and c.1552G>A (p.Gly518Arg)—alongside two intronic variants affecting splicing: c.1424–4C>G, which alters the guanine nucleotide at position—4 of exon 22, and c.4529-2A>T, located at the canonical splice acceptor site of intron 49. These variants were identified in three Chinese families with XLAS caused by mutations in the *COL4A5* gene [101].

Patient P07 (C.M.) is a 24-year-old male diagnosed with Stage IV-V CKD, presenting with persistent proteinuria and glomerular hematuria (acanthocytes). His clinical history is notable for childhood-onset bilateral sensorineural hearing loss and ocular involvement -including myopia, astigmatism, and dot-and-fleck retinopathy—a triad highly suggestive of Alport Syndrome. The initial manifestations appeared at age 4, with macroscopic hematuria and albuminuria. Additional symptoms include a chronic cough and recurrent wheezing. Importantly, the patient was adopted, and therefore no biological family medical history is available.

Sequence analysis using the Blueprint Genetics (BpG) Alport Syndrome Panel identified a hemizygous missense *COL4A5* c.2714G>A, p.(Gly905Asp) variant which is associated with XLAS. This variant is absent in gnomAD v2 database. It has been previously been reported in three patients with Alport syndrome, including a de novo occurrence in a female patient (PMID: 22921432, 30477285, 30577881) [101,102,103].

In silico prediction using the REVEL computational tool suggests that this variant is disease-associated. This mutation affects a highly conserved glycine residue located within the triple helical domain of this fibrillar collagen. The substitution of glycine residues in the collagen triple helix represents a well-established pathogenic mechanism in this type of network collagen, as documented in the HGMD [81]. Based on these findings and in accordance with the ACMG guidelines, the variant has been classified as likely pathogenic [99].

Patient P08 (C.C.) is a 47-year-old male diagnosed with Stage IV CKD, confirmed by renal biopsy showing global glomerulosclerosis. Clinical manifestations include bilateral renal cysts, microscopic hematuria, marked proteinuria (>2g/24h), and hypertension, in the absence of hearing impairment. Notably, he has two sons, one of whom exhibits symptoms suggestive of Alport syndrome.

Genetic analysis using the Blueprint Genetics (BpG) FLEX Cystic Kidney Disease Panel identified a heterozygous likely pathogenic in-frame insertion in the *COL4A3* gene (c.3546_3548dup, p.(Gly1183dup)), associated with Autosomal Dominant Alport Syndrome (ADAS). The patient also carries a heterozygous intronic deletion in the *MYH9* (c.5275-60_5275-11del) gene, currently classified as a Variant of Uncertain Significance (VUS).

Pathogenic variants in the *MYH9* gene are responsible for an autosomal dominant disorder characterized by macrothrombocytopenia and granulocyte inclusions, which may or may not be accompanied by nephritis or sensorineural hearing loss. This condition is known as MYH9-related disease (MYH9-RD) and corresponds to the clinical entry MATINS in the OMIM database (OMIM 155100) [9]. Although historically classified as four distinct entities—May–Hegglin anomaly (MHA), Sebastian syndrome (SBS), Fechtner syndrome (FTNS), and Epstein syndrome (EPSTNS) [9]—MYH9-RD is now recognized as a single clinical spectrum with variable expressivity [104]. In addition, specific mutations in *MYH9* have also been associated with autosomal dominant deafness-17 (DFNA17; OMIM 160775) [9].

Although the patient (P08) exhibited normal platelet levels on complete blood count (CBC), ongoing hematological monitoring was advised. Genetic testing of his son, who presented with renal manifestations (recurrent proteinuria and hematuria) without thrombocytopenia, revealed the same two genetic variants found in the father, confirming a diagnosis of Alport syndrome. As a result, nephrological and hematological follow-up, as well as periodic audiometric assessments, were recommended for the son as well.

The patient’s second son, who exhibited no renal manifestations but was found to have asymptomatic thrombocytopenia, underwent genetic testing that identified the *MYH9* c.5275-60_5275-11del variant (VUS). This variant was also present in the father; however, the son did not carry the *COL4A3* mutation. Consequently, periodic hematological monitoring—including a complete blood count (CBC) and peripheral blood smear—was recommended.

Patient P09 (S.A.) is a 17-year-old male diagnosed at age 13 with insulin-dependent diabetes mellitus (suspected MODY), obesity, stage II CKD, hepatocytolysis (with positive IgG anti-HEV antibodies), and an emotional disorder. Renal ultrasonography revealed multiple bilateral cortical and corticomedullary cysts of varying sizes, while biochemical markers showed mild azotemia in the absence of hematuria or proteinuria. Given the clinical triad of diabetes, renal cystic disease, and hepatic involvement, HNF1B-MODY syndrome—also known as MODY5 or Renal Cysts and Diabetes Syndrome (RCAD) (OMIM 137920) [9]—is strongly suspected. Genetic testing has been recommended to confirm the diagnosis.

Copy number variant (CNV) analysis using the Blueprint Genetics (BpG) Whole-Exome identified a heterozygous 1.9 Mb deletion on chromosome 17: seq[GRCh37]del(17)(q12q12); chr17:g.34160776_36093874del. This deletion encompasses the exons 3 through 9 of the *HNF1B* gene. Given that *HNF1B* haploinsufficiency represents the primary mechanism underlying RCAD syndrome (MODY5), this genomic alteration is considered diagnostic and fully consistent with the patient’s clinical presentation.

This deletion spans approximately 1.9 Mb within the genomic region 17:34160776-36093874 and includes 35 protein-coding genes. Among these, four are clinically significant according to the OMIM database: *HNF1B* (OMIM 189907), associated with autosomal dominant inheritance, and *ACACA* (OMIM 200350), *PIGW* (OMIM 610275), and *ZNHIT3* (OMIM 604500), each linked to autosomal recessive disorders [9].

At the molecular level, this deletion results in an out-of-frame loss of exons 3–9 of the *HNF1B* gene, which is predicted to disrupt the open reading frame. Of note, similar deletions affecting this region have been reported only rarely in control cohorts, including four cases in the Exome Aggregation Consortium (ExAC) [105] and three in the gnomAD SVs v2.1 database [79].

According to HGMD Professional database (2024.3), 74 distinct gross deletions involving the *HNF1B* gene at the 17q12 locus have been documented [81]. Furthermore, the DECIPHER database records over 110 patients with overlapping deletions in this region [106]. Notably, a deletion of this exact genomic size has not been previously reported in the medical literature or specialized variant databases.

Deletions within the 17q12 region are the underlying cause of Renal Cysts and Diabetes Syndrome (RCAD), frequently referred to as 17q12 microdeletion syndrome (OMIM 614527; GeneReviews NBK401562) [9,107,108]. This recurrent deletion typically spans 1.2–1.4 Mb and encompasses the *HNF1B* gene. Phenotypically, the syndrome is characterized by a highly variable clinical presentation, primarily involving a triad of features: renal malformations (such as CAKUT and tubulointerstitial disease), maturity-onset diabetes of the young (MODY5), and a spectrum of neurodevelopmental or neuropsychiatric conditions, including autism spectrum disorder, schizophrenia, and intellectual disability (GeneReviews NBK401562) [107].

Diseases caused by *HNF1B* variants follow an autosomal dominant inheritance pattern. In the case of patient P09, both parents are clinically healthy, suggesting that the mutation likely arose de novo; however, germline mosaicism in one of the parents cannot be entirely excluded. Should the patient have offspring, each child would have a 50% risk of inheriting the variant and developing the condition.

Patient P10 is a 4-year-old male, born prematurely at 28 weeks to consanguineous parents. The pregnancy was complicated by polyhydramnios, and his neonatal course was marked by cerebral hemorrhage and acute renal failure. At two years of age, he developed a urinary tract infection caused by extended-spectrum beta-lactamase (ESBL)-producing *Escherichia coli*. Notably, a dimercaptosuccinic acid (DMSA) scintigraphy performed following the infection showed no evidence of post-infectious renal scarring.

Clinically, the patient presents with polyuria, polydipsia, and growth failure. These findings, together with biochemical abnormalities including hyposthenuria, hyponatremia, hypokalemia, hypercalciuria, and metabolic alkalosis, as well as the presence of nephrocalcinosis, are highly suggestive of Bartter syndrome. Additionally, cranial computed tomography (CT) revealed ventriculomegaly and hypoplasia of the corpus callosum.

Sequence analysis using the Blueprint Genetics (Bp) Whole-Exome Plus identified three homozygous missense variants: *SLC12A1* c.2755G>C, p.(Asp919His), *POLG* c.1760C>T, p.(Pro587Leu) and *POLG* c.752C>T, p.(Thr251Ile).

The *SLC12A1* gene (OMIM 600839) encodes the kidney-specific sodium-potassium-chloride cotransporter NKCC2, which is located on the luminal membrane of renal epithelial cells in the thick ascending limb of Henle’s loop and the macula densa. This transporter plays a critical role in urine concentration by mediating a substantial portion of sodium, potassium, and chloride reabsorption. Through alternative splicing, the gene generates multiple transcript variants that encode distinct protein isoforms.

Pathogenic variants in *SLC12A1* are associated with Bartter syndrome type 1 (BARTS1; OMIM 601678) [9]. According to the HGMD Professional database (2023.1), 117 disease-causing variants have been reported in this gene in association with Bartter syndrome, including 61 missense, 17 nonsense, 7 splice-site, 30 small insertions or deletions, and two gross deletions [81].

The *SLC12A1* variant c.2755G>C (p.Asp919His) has been classified as likely pathogenic. This classification is supported by several lines of evidence: a well-established gene–phenotype correlation, its extreme rarity in control populations, consistent in silico predictions of pathogenicity, and its identification in the homozygous state in individuals with similar clinical presentations. The patient’s homozygous genotype is consistent with autosomal recessive inheritance. As both parents are confirmed obligate carriers, each sibling has a 25% risk of being affected (i.e., homozygous for the variant). Accordingly, genetic counseling and cascade testing for family members were recommended.

In patient P10, genetic analysis also identified a homozygous complex variant in the *POLG* gene: c.[752C>T;1760C>T], resulting in the protein changes p.(Thr251Ile) and p.(Pro587Leu). Both individual variants are classified as pathogenic, and their presence in a homozygous state is consistent with an autosomal recessive inheritance pattern for *POLG*-related mitochondrial disorders.

The *POLG* gene (OMIM 174763) encodes DNA polymerase gamma, the enzyme essential for mitochondrial DNA (mtDNA) synthesis [9]. Pathogenic variants in this gene can severely impair polymerase activity, leading to mtDNA depletion or the accumulation of multiple mtDNA deletions—both of which result in systemic mitochondrial dysfunction. Consequently, *POLG*-related disorders encompass a broad and overlapping spectrum of neurological phenotypes that manifest across the lifespan, from early childhood to late adulthood [109].

The *POLG* mutational landscape is characterized by significant allelic heterogeneity. The HGMD Professional database (v2023.2) documents over 350 disease-causing variants [81], while the NCBI ClinVar database lists more than 300 pathogenic or likely pathogenic entries [80]. Although the mutational spectrum includes splicing defects, small indels, and gross deletions, missense mutations remain the most prevalent. Among these, the p.(Ala467Thr) and p.(Trp748Ser) variants are the most frequently identified, acting as pleiotropic markers associated with a diverse range of clinical phenotypes (Human DNA Polymerase Gamma Mutation Database) [110].

According to gnomAD, the *POLG* c.1760C>T (p.Pro587Leu) variant is present in 433 heterozygous individuals and one homozygous individual, while the *POLG* c.752C>T (p.Thr251Ile) variant is found in 434 heterozygotes and one homozygote [79]. These two variants are frequently documented together on the same chromosome (in *cis*), forming the complex allele *POLG* c.[1760C>T;752C>T], p.[Pro587Leu;Thr251Ile], as registered in ClinVar under Variation IDs 13503 and 13505 [80].

This complex allele was first described by Van Goethem et al. [110] in two sisters who presented at age 15 with gastrointestinal symptoms. The variant was identified in *trans* with a pathogenic *POLG* c.2591A>G (p.Asn864Ser) variant. Additional manifestations included progressive external ophthalmoplegia (PEO), axonal sensory ataxic neuropathy, muscle weakness, and cachexia (PMID: 12825077) [111].

Since then, the *POLG* c.[1760C>T;752C>T], p.[Pro587Leu; Thr251Ile] complex variant has been documented in numerous patients and families across a broad spectrum of *POLG*-related phenotypes. In the majority of reported cases, it occurs in conjunction with a second pathogenic *POLG* variant, as supported by multiple studies in the literature (PMID: 25660390, 30936349, 18828154, 19578034) [112,113,114,115].

At least three patients harboring the homozygous complex allele have been reported in the literature, each presenting with a broad phenotypic spectrum (PMID: 21138766, 27538604, 29474836) [116,117,118]. These include a case of Alpers-Huttenlocher syndrome (PMID: 21138766) [116], a patient with ptosis and dysphagia (PMID: 27538604) [117], and an individual diagnosed with SANDO (sensory ataxic neuropathy, dysarthria, and ophthalmoparesis) accompanied by movement disorders and depression (PMID: 29474836) [118]. Clinical observations suggest that this homozygous complex allele is associated with reduced penetrance and variable expressivity, typically resulting in a milder or later-onset phenotype compared to other pathogenic *POLG* mutations [9,109].

A multidisciplinary follow-up plan—including nephrological, neurological, and pediatric evaluations—was recommended for patient P10. Crucially, the administration of valproic acid must be avoided, as pathogenic variants in the *POLG* gene are strongly associated with an increased risk of drug-induced liver toxicity [109].

Given that the proband is homozygous for all three identified variants, it is highly likely that both parents are asymptomatic carriers. Therefore, molecular testing of the parents was advised to confirm their carrier status and to assess the recurrence risk for future pregnancies.

### 3.2. Genetic Testing in Genetic Syndromes Associated with CKD—An Important Pillar of Precision Medicine in Nephrology

Advances in molecular genetics have significantly deepened our understanding of the genetic architecture underlying chronic kidney disease (CKD). The widespread implementation of whole-exome sequencing and targeted gene panels has helped bridge the gap between clinical presentation and molecular diagnosis. As a result, the ability to identify pathogenic variants has laid the foundation for personalized management strategies in patients with genetic syndromes associated with CKD.

To improve clinical outcomes in suspected cases of Alport syndrome or steroid-resistant nephrotic syndrome (SRNS), the literature supports performing targeted genetic testing as early as possible in the diagnostic process [119,120]. This approach not only aids in patient stratification but also has a direct impact on therapeutic choices.

The ongoing shift toward Whole-Genome Sequencing (WGS) heralds a new era in precision nephrology, enabling the identification of novel variants that can guide personalized interventions—such as optimizing or discontinuing immunosuppressive therapies—and enhance the strategic planning of post-transplant immunosuppression [121,122].

In accordance with the Kidney Disease Improving Global Outcomes (KDIGO) guidelines, genetic testing is recommended for all patients with CKD when a genetic cause is suspected, regardless of age [65]. This approach proves especially valuable in cases of advanced disease where the underlying etiology remains unclear, and renal biopsy is either not feasible or inconclusive [123,124]. Identifying a pathogenic variant in an index patient also enables early intervention in at-risk relatives through cascade screening [125]. Ultimately, the integration of genomics into routine nephrology practice has the potential to streamline diagnosis, reduce the need for invasive procedures, and optimize therapeutic outcomes [125,126,127].

### 3.3. Genetic Counseling in Patients with Genetic Syndromes Associated with CKD

Identifying a positive family history and providing genetic counseling significantly enhance diagnostic precision and optimize therapeutic outcomes for patients with genetic syndromes associated with CKD [124].

In the analyzed cohort, molecular genetic testing improved diagnostic accuracy for patients presenting with symptoms suggestive of a genetic syndrome associated with CKD (P01, P03–P10). Notably, the utility of testing was maintained even in the absence of a positive family history (P01, P07, P09, and P10), underscoring the importance of genetic screening in sporadic cases with a suggestive clinical phenotype.

We consider that these findings highlight the value of molecular genetic testing as a first-line diagnostic tool, rather than merely a confirmatory method.

Genetic testing also played an essential role in the presymptomatic diagnosis of patient P02, who was asymptomatic at the time of initial evaluation, in the context of a positive family history. Conversely, the absence of a family history can mislead clinicians and significantly complicate the diagnostic process for genetic syndromes associated with CKD, often leading to diagnostic delays. Modern NGS-based genetic testing has helped overcome these challenges by enabling a highly accurate molecular diagnosis, even in the absence of previous familial cases.

Comprehensive genetic counseling was provided to all participants and their families. Based on the specific monogenic inheritance pattern identified, the management plan included recommendations for genetic testing of symptomatic relatives for the familial variant, as well as ongoing renal monitoring—such as ultrasound, urinalysis, and blood tests—for at-risk family members.

These findings underscore the value of genetic testing in refining diagnoses, enabling the identification of presymptomatic carriers, and guiding familial screening—even in the absence of a clear family history of the disease.

A major benefit of genetic diagnosis in our study cohort, which consisted predominantly of pediatric patients, was the increased diagnostic accuracy and the potential to avoid invasive renal biopsies.

A primary limitation of this study is its small sample size, which restricts the generalizability of our findings to the broader population of patients with suspected genetic syndromes and CKD in the Moldova region. This limitation largely stems from limited access to comprehensive molecular testing, driven by high costs—often borne by patients’ families—and the inherent complexity of genomic data interpretation. In addition, incomplete family histories and a lack of precise genealogical data in some cases hindered a thorough clinical anamnesis.

Despite these challenges and the well-documented population-specific variability of genetic variants, our findings remain consistent with those reported in larger cohorts of patients with genetic syndromes associated with CKD.

In Romania, the primary source of official data on CKD epidemiology is the Romanian Renal Registry (RRR). However, this registry collects information exclusively from patients with ESRD—specifically those undergoing dialysis or transplantation. While a Predialysis Registry for stages 1–5 was initiated in 2019, its coverage is incomplete as it relies on reports that do not encompass the entire asymptomatic patient population. As a result, early-stage CKD (stages 1–2) remains underdiagnosed, particularly in children, due to its often subtle or asymptomatic presentation. Furthermore, specific statistical data on the incidence of CKD in the pediatric population are scarce and are typically either aggregated within general population reports or derived from targeted epidemiological studies.

To the best of our knowledge, no studies to date have investigated the genetic etiology or the spectrum of genetic variants—including those associated with genetic syndromes involving CKD—in the Romanian population affected by this condition.

In this context, although our study includes a limited number of cases, it may serve as a foundational step for future national research involving both pediatric and adult populations with CKD. Identifying risk allelic variants specific to the Romanian population could facilitate the implementation of preventive strategies, such as genetic screening for populations at high risk of developing CKD, including those with genetic syndromes associated with the disease.

## 4. Materials and Methods

We retrospectively analyzed a group of ten patients (including their families) who were diagnosed via molecular testing (Next-generation sequencing, NGS) with a genetic syndrome associated with CKD. Genetic testing was performed both to confirm the diagnosis in symptomatic patients and for presymptomatic diagnosis in asymptomatic individuals with a positive family history. In these cases, the identification of the pathogenic variant also aimed to establish a management plan that included monitoring renal function, early detection of renal symptoms, and, in correlation with these findings, personalized treatment.

All patients originated from the same geographical region (Moldova area) and were registered at the Nephrology Clinic and the Regional Center for Medical Genetics Iași of the Sf Maria Children’s Emergency Hospital Iași, Romania. Diagnosis confirmation was achieved through extended genetic testing—targeted gene panels or Whole-Exome Sequencing at the Blueprint Genetics (BpG, Espoo, Finland) laboratory using Next-Generation Sequencing (NGS) (Illumina technology, San Diego, CA, USA) or testing of a targeted gene panel using the TruSightCardio Illumina kit (Genomic Medicine Center Laboratory, Timisoara, Romania).

For the WES analysis, the total genomic DNA was extracted from the biological sample using a bead-based method. The quantity of DNA was assessed using a fluorometric method. After the assessment of DNA quantity, the qualified genomic DNA sample was randomly fragmented using non-contact, isothermal sonochemistry processing. A sequencing library was prepared by ligating sequencing adapters to both ends of DNA fragments. Sequencing libraries were size-selected with a bead-based method to ensure the optimal template size and amplified by polymerase chain reaction (PCR). Regions of interest (exons and intronic targets) were targeted using a hybridization-based target capture method. The quality of the completed sequencing library was controlled by ensuring the correct template size and quantity and to eliminate the presence of leftover primers and adapter–adapter dimers. Ready sequencing libraries that passed the quality control were sequenced using Illumina’s sequencing-by-synthesis method with paired-end sequencing (2 × 150 bases). The primary data analysis converting images into base calls and associated quality scores was carried out by the sequencing instrument using Illumina’s proprietary software v2.20 (bcl2fastq), generating CBCL files as the final output. For bioinformatics and quality control, base-called raw sequencing data were transformed into FASTQ format using Illumina’s software v2.20 (bcl2fastq). Sequence reads of each sample were mapped to the human reference genome (GRCh37/hg19). Burrows–Wheeler Aligner (BWA-MEM) software was used for read alignment. Variant data were annotated using a collection of tools (VcfAnno and VEP) with a variety of public variant databases, including but not limited to gnomAD, ClinVar, and HGMD. The patient’s sample was subjected to thorough quality control measures, including assessments for contamination and sample mix-up. Copy number variations (CNVs), defined as single exon or larger deletions or duplications (Del/Dups), were detected from the sequence data using a proprietary bioinformatics pipeline. The pathogenicity potential of the identified variants was assessed by considering the predicted consequence, the biochemical properties of the codon change, the degree of evolutionary conservation, as well as a number of reference population databases and mutation databases, such as but not limited to the 1000 Genomes Project, gnomAD, ClinVar, and HGMD Professional. For missense variants, in silico variant prediction tools such as SIFT, PolyPhen, and MutationTaster were used to assist with variant classification. In addition, the clinical relevance of any identified CNVs was evaluated by reviewing the relevant literature and databases such as the 1000 Genomes Project, Database of Genomic Variants, ExAC, gnomAD, and DECIPHER.

The pathogenicity of the identified gene variants was assessed according to the American College of Medical Genetics and Genomics and Association for Molecular Pathology (ACMG/AMP) guidelines [99]. For the interpretation of the variants identified in patients with CKD, we also used the HGMD Professional [81] and ClinVar databases [80].

In the case of patient P06, the diagnosis of Alport syndrome was confirmed by sequencing a panel of 4813 genes using the Illumina TruSight Cardio Sequencing Panel kit, in the Research Laboratory of the Timișoara Genomic Medicine Center. This panel also includes candidate genes for CKD (eg *COL4A5*).

The list of genes covered by the panel can be consulted at: https://support.illumina.com/downloads/trusight_one_sequencing_panel_product_file.html, (accessed on 31 January 2026)

It was used the NGS technique using Illumina’s MiSeq platform, which allows the analysis of coding sequences from genomic DNA. In the first step, genomic DNA fragmentation was performed, followed by the amplification of coding sequences and the generation of libraries using the Illumina TruSight Cardio Sequencing Panel kit. End-to-end bioinformatics algorithms were implemented, including nitrogenous base alignment, primary filtering of low-quality reads and likely artifacts, and variant annotation, using Isis (Analysis Software) 2.5.1.3; BWA (Aligner) 0.6.1-r104-tpx; SAMtools 0.1.18 (r982:295); Annovar (Variant Caller) 1.7. The data analysis was performed at the level of current knowledge, using the databases: UCSC (University of California Santa Cruz Genomisc Institute) Genome Browser, OMlM (Online Mendelian Inheritance in Man), DGV (Database of Genomic Variants). All disease-causing variants reported in HGMD^®^, ClinVar (class 1), as well as all variants with minor allele frequency (MAF) less than 1% in the ExAc database were considered. Evaluation was focused on exons. All transmission patterns were considered, taking into account family history and clinical information Reported variants were correlated with clinical phenotype. The variants were interpreted according to the American College of Medical Genetics and Genomics and Association for Molecular Pathology (ACMG/AMP) guidelines. Mendelian variants were also classified according to ACMG: Pathogenic/Likely pathogenic/Variant of uncertain significance (VUS)/Likely benign/Benign [99].

We analyzed the data obtained through family history—including the family tree for each patient and their family—and established correlations between the identified genetic variant and the corresponding phenotype.

The results were compared with data from the specialized literature, highlighting the particularities of each patient within the context of their family.

The study was conducted according to the Helsinki II declaration and was approved by the Ethics Committee of the Children’s Emergency Clinical Hospital, Sf. Maria Iași, Romania (Certificate no. 43103/2025).

Informed consent from both parents was obtained for each child included in our study, as well as for all adults who were clinically evaluated and who underwent genetic testing.

## 5. Conclusions

Rare hereditary kidney diseases and genetic syndromes associated with CKD represent significant challenges in modern nephrology. However, recent advances in molecular technology, combined with broader access to genetic testing and the growing potential for targeted therapies, have generated considerable interest in this field. Emerging evidence suggests that precision medicine can significantly improve patient care by enabling more accurate diagnostic approaches.

The implementation of advanced genetic tools—such as targeted gene panels, Whole-Exome Sequencing (WES), and Whole-Genome Sequencing (WGS)—enables precise molecular diagnoses, leading to improved prognostic accuracy and the development of personalized therapeutic strategies. Moreover, a molecular-first approach may obviate the need for invasive renal biopsies, particularly in cases where histological findings are inconclusive. The identification of specific allelic variants is also essential for genetic counseling and for screening at-risk family members, playing a critical role in informed decision-making regarding living donor kidney transplantation.

Comprehensive genetic testing not only enables the identification of known variants but also facilitates the discovery of novel ones, contributing to a deeper understanding of the complex molecular architecture underlying the etiology, morbidity, and mortality associated with CKD. Future research involving large patient cohorts may help elucidate regional differences in CKD prevalence based on specific allelic profiles. Beyond establishing genotype–phenotype correlations, such studies offer a valuable opportunity to investigate the factors driving phenotypic variability—explaining why patients with the same mutation may present with different degrees of disease severity. As testing costs continue to decline and accessibility improves, genetic analysis is expected to become a routine diagnostic tool in standard nephrology practice.

## Figures and Tables

**Table 1:** Genetic Heterogeneity in Syndromes Associated with Chronic Kidney Disease.

Disease	Gene/Locus	Locus	Inheritance	Syndrome	OMIM [9]	Bibliography
Growth and structural abnormalities						
CAKUT	*GREB1L*	18q11.1-q11.2	AD	Renal hypodysplasia/aplasia 3	617805	[10,11,12,13]
	*PAX2*	10q24.31	AD	Glomerulosclerosis, focal segmental, 7; Papillorenal syndrome	616002: 120330	[14,15]
	*HNF1B*	17q12	AD	Renal cysts and diabetes syndrome	137920	[16,17]
ADPKD	*PKD1*	16p13.3	AD	Polycystic kidney disease 1	173900	[18,19]
	*PKD2*	4q22.1	AD	Polycystic kidney disease 2	613095	[19,20,21]
Bardet Biedl Syndrome	*BBS*		AR	Bardet Biedl (BBS1-26)		[22,23]
TSC	*TSC1*	9q34.13	AD	Tuberous sclerosis-1	605284	[24,25]
	*TSC2*	16p13.3	AD	Tuberous sclerosis-2	613254	[24,25]
Glomerular diseases						
Syndromic FSGS	*WT1*	11p13	AD	Frasier syndrome: Denys–Drash syndrome	607102	[10,11]
	*LMX1B*	9p33.3	AD	Focal segmental glomerulosclerosis 10: Nail-patella syndrome	602575	[10,26,27]
	*LAMB2*	3p21.31	AR	Pierson syndrome (PIERS)	609049	[10]
	tRNALeu(UUR)	mDNA		MELAS	590050	[10,11,28,29]
	*COQ2*	4q21.23	AR	Coenzyme Q10 deficiency, primary, 1	607426	[11]
	*ITGB4*	17q25.1	AR	Epidermolysis bullosa	619816	[11,30]
	*MYH9*	22q12.3	AD	Epstein–Fechtner syndrome	155100	[10,31,32]
Alport syndrome	*COL4A3*	2q36.3	AD	Alport syndrome 3A, autosomal dominant	120070	[33,34]
	*COL4A4*	2q36.3	AR	Alport syndrome 2, autosomal recessive	203780	[33,35]
	*COL4A5*	Xq22.3	XLR	Alport syndrome 1, X-linked	303630	[33,34]
Tubular diseases						
Barter Syndrome	*CLCNKB*	1p36.13	AR	Bartter syndrome, type 3 (BARTS3)	602023	[36,37]
	*SLC12A1*	15q21.1	AR	Bartter syndrome, type 1 (BARTS1)	601678	[38,39,40]
	*KCNJ1*	11q24.3	AR	Bartter syndrome, type 2 (BARTS2)	241200	[9,40]
	*BSND*	1p32.3	AR	Bartter syndrome, type 4a (BARS4A)	602522	[40,41,42]
	*CLCNKA*/ *CLCNKB*	1p36.13	DR	Bartter syndrome, type 4b, digenic (BARS4B)	613090	[36,37,43]
	*MAGED2*	Xp11.21	XLR	Bartter syndrome, type 5, antenatal, transient	300971	[9,40]
Gitelman syndrome	*SLC12A3*	16q13	AR	Hypomagnesemia-hypokalemia, primary renotubular, with hypocalciuria syndrome	263800	[38,39,40,41,42,43,44]
Cystinuria	*SLC3A1*	2p21	AR/AD	Cystinuria type A	104614	[45,46]
	*SLC7A9*	19q13.11	AR/AD	Cystinuria Type B	604144	[45,47]
Metabolic diseases						
Cystinosis	*CTNS*	17p13.2	AR	Cystinosis, nephropathic	606272	[48]
Fabry disease	*GLA*	Xq22.1	XL	Angiokeratoma corporis diffusum Fabry disease	300644	[49,50]
Hyperoxaluria	*AGXT*	2q37.3	AR	Type I primary hyperoxaluria (HP1)	259900	[51]

ADPKD: Autosomal dominant polycystic kidney disease; AD: autosomal dominant, AR: autosomal recessive; XLR: X-linked recessive; DR: Digenic, recessive: CAKUT: Congenital abnormalities of the kidney and urinary tract; FSGS: Focal and segmental glomerulosclerosis; TSC: Tuberous Sclerosis Complex; BARS4A: Neonatal Bartter syndrome type 4A with sensorineural deafness; BARS4B: Neonatal Bartter syndrome type 4B with sensorineural deafness (digenic); MELAS: Myopathy, encephalopathy, lactic acidosis, and stroke-like episodes; mDNA: mitochondrial DNA.

**Table 2:** Genetic Heterogeneity Correlated with Phenotypic Variability in Patients with Genetic Syndromes Associated with CKD.

Patient ID	Mutation	Locus	OMIM	Transcript	Protein	Inheritance Pattern	Genotype	Effect of the Mutation/Pathogenicity	Variant Previously Reported	Syndrome
P01 (V.L)	*PKD1* c.6040C>T	16p13.3	173900	NM_001009944.3	p.(Gln2014*)	AD	Hz	Nonsense/P	known	Polycystic kidney disease 1
P02 (B. A)	*PKD1* c.9005C>A	16p13.3	173900	NM_001009944.3	p.(Ser3002*)	AD	Hz	Nonsense/LP	known	Polycystic kidney disease 1
P03 (M.A)	*PKD1* c.1405_1411del	16p13.3	173900	NM_00100944.3	p(Gly469Argfs*87)	AD	Hz	Frameshift/LP	new	Polycystic kidney disease 1
P04 (C.A)	*PKD2* c.916C>T	4q22.1	613095	NM_000297.4	p.(Arg306*)	AD	Hz	Stop_gained (nonsense)/P	known	Polycystic kidney disease 2
P05 (L.E)	*COL4A5* c.2587G>T	Xq22.3	303630	NM_000495.5	p.(Gly863Cys)	XLD	Hz	Missense/LP	new	Alport
P06 (P.V.)	*COL4A5* c.4993A>T	Xp22.3	303630	NM_033380.3	p.(Ser1665Cys)	XLD	Hmz	Missense/LP	new	Alport
P07 (C.M.)	*COL4A5* c.2714G>A	Xp22.3	134797	NM_000495.5	p.(Gly905Asp)	XLD	Hmz	Missense/LP	known	Alport
P08 (C.C)	*COL4A3* c.3548dup	2q36.3	134797	NM_000138.4	p.(Gly1183dup)	AD	Hz	Inframe insertion/LP	new	Alport
P09 (S.A)	*HNF1B* seq[GRCh37] del(17)(q12q12); chr17:g.34160776_36093874del	15q21.1	189907			AD	Hz	Deletion/P	Known	MODY5
P10 (A.C)	*SLC12A1* c.2755G>C	15q21.1	600839	NM_000338.3	p.(Asp919His)	AR	Ho	Missense/LP	Known	Bartter Syndrome type 1

MODY5: Maturity-onset diabetes of the young, type 5; Hmz: Hemizygouse genotype; Hz: Heterozygouse genotype; Ho: Homozygouse genotype; *PKD1*: Polycystic kidney disease 1; *PKD2*: Polycystic kidney disease 2; P: Pathogenic: LP: Likely pathogenic; AD: Autosomal dominant; AR: Autosomal recessive; XLD: X-linked dominant.

**Table 3:** Clinical and Paraclinical Data of Patients with Genetic Syndromes Associated with CKD **.

Criteria (Normal Value)	P01 (V.L)	P02 (B.A)	P03 (M.A)	P04 (C.A)	P05 (L.E)	P06 (P.V)	P07 (C.M)	P08 (C.C)	P09 (S.A.)	P10 (A.C.)
Age * (years/months)	1y 10mo	17 mo	13y	15 y	10y	26 y	24 y	47 y	17 y	4 y
Gender (M/F)	F	F	F	M	M	M	M	M	M	M
Family history of CKD	−	+	+	+	+	+	−	+	−	−
Creatinine (0.15–0.29 mg/dL)	0.22	0.19	0.55	0.3	0.56	0.49	3.77	0.7	1.47	0.65
Urea (19.18–47.35 g/dL)	38	24	33	24	33	52	108	33	81	44
Uric acid (2.20–5.59 mg/dL)	3.2	2.5	3.6	3.5	5	4.9	7.8	6.2	10.8	5.6
eGFR (>60 mL/min/1.73 m^2^) (91.5 ± 17.8 mL/min/1.73 m^2^)	105	145	87	108	135	136	20	54	69	99
Microscopic hematuria	+	−	+	+	+	+	+	+	−	−
Macroscopic hematuria	−	−	−	-	+	−	+	−	−	−
Proteinuria/24 h (0–300 mg/L)	99	−	423	200.2	199	+	+	+	<150	+
Total protein (57–82 g/L)	76	65	73.7		68.2	72.6	50	72.1	71.8	6.9
UPCR (<2)	-	-	-	-	0.26–0.32	-	-	-	-	-
Hemoglobin (11.3–14.1 g/dL)	9.6	12.1	13.7	9.5	10.2	14.5	10.8	15.6	16.1	12.6
Ferritin (15–150 µg/L)	35.21	45	-	-	70.6	17.85	-	-	106.7	
Ferrum seric (16.2–164.8 µg/dL)	53	57	-	52	64	-	-	-	159	
Triglycerides (44–197 mg/dL)	-	-	45	50	89	-	150	74	144	
Total Cholesterol (112–208 mg/dL)	145	120	159	180	220	-	250	135	155	
Total Calcium (8.4–10.2 mg/dL)	-	-	9.7	-	-	11	-	9.8	10.3	10.1
Phosphorus (3.2–5.9 mg/dL)	3.6	4.9	4.2	-	-	-	-		3.4	3.9
Alkaline phosphatase (113–438 U/L)	120	138	91	-	-	-	-	136	215	230
Fibrinogen (2–4 µmol/L)	2	2	3	-	2.5		-	2.39	3	-
C-Reactive Protein (0–5 mg/L)	1.3	1	0.5	-	1.3	0.78	12	0.72	0.6	1.1
Serum chloride (103–112 mmol/L)	-	-	-	-	-	-	-	-	-	95.4
Serum potassium (3.87–5.4 mmol/L)	-	-	-	-	-	-	-	-	-	3.1
Serum sodium (139–146 mmol/L)	-	-	-	-	-	-	-	-	-	138
Calciuria (100–300 mg/24 h)	-	-	-	-	-	-	-	-	<25	216.3 mg/24 h = 16 mg/kg/24 h
Diabetes mellitus	-	-	-	-	-	-	-	-	+	-
Hypertension	-	-	-	-	-	-	+	-	-	-
Renal Ultrasound	cysts	cysts	cysts	cysts, horseshoe kidney	-	-	-	-	cysts	Hydronephrosis
Renal biopsy	-	-	-	-	-	-	-	-	-	-
BMI (kg/m^2^)	18.89	18.2	19.3	19.7	13.6	16.6	17.3	19.62	26.2	15.3

* Age (y/mo): The age of the patients (years/months) at the time of diagnosis; eGFR: Glomerular Filtration Rate; UPCR: Urine Protein Creatinine Ratio. ** The values of the biochemical parameters are those recorded at the time of diagnosis; subsequently all patients were monitored and evaluated periodically (renal ultrasound and biochemical parameters); BMI: Body mass index.

## Data Availability

The original contributions presented in this study are included in the article. Further inquiries can be directed to the corresponding author.

## Abbreviations

The following abbreviations are used in this manuscript:

CKD	Chronic Kidney Disease
ADPKD	Autosomal Dominant Polycystic Kidney Disease
ADTKD	Autosomal dominant tubulointerstitial renal disease
XLD	X-linked Dominant
AD	Autosomal Dominant
AR	Autosomal recessive
ESRD	End-stage renal disease
RRT	Renal Replacement Therapy
XLAS	X-linked Alport Syndrome
ADAS	Autosomal dominant Alport syndrome
SNHL	Sensorineural hearing loss
